# The Potency Question

**Published:** 1894-11

**Authors:** 


### HOMŒOPATHIC PHYSICIAN,

A MONTHLY JOURNAL OF

HOMŒOPATHIC MATERIA MEDICA AND CLINICAL MEDICINE.

"If our school ever gives up the strict inductive method of Hahnemann, we are lost, and deserve only to be mentioned as a caricature in the history of medicine."—CONSTANTINE HERING.

Vol. XIV. NOVEMBER, 1894. No. 11.

### EDITORIAL.

THE POTENCY QUESTION.—The leading article in this month's number of THE HOMŒOPATHIC PHYSICIAN, by the venerable Dr. Selfridge, of California, entitled "Medicine," is a timely exposition of the position of Homœopathy before the world.

"The truth or falsity of Homœopathy is susceptible of demonstration," says the writer of the article referred to, "and yet, in the light of this nineteenth century, with the experiences of past decades spread out before them, with an abundance of opportunities to verify or disprove, if they can, the statements of Hahnemann, there are hundreds of physicians who, without examination, pronounce Homœopathy a delusion and a snare." Yes, and these men say it in the journals avowedly homœopathic, but omit the action suitable to such words of quitting the fellowship of the school. Instead, they stand before the world as homœopathists, and continue their warfare against the school in the journals. Thus they afford an abundance of material for the old-school doctors who, whenever they wish to fire a shot or two at our school, go to our magazines for their ammunition.

"To be a good homœopathic physician does not necessarily require him to be a high-potency man. On the contrary, his practice may be strictly in accordance with the law of similars, even though he administer the mother tincture, and *vice versa*. A physician may use only the high, or even the highest potencies, and not be a practitioner of Homœopathy, for the simple reason that he does not select his remedy in accordance with the law of similars."

This plain proposition, so concisely stated, does not seem to be understood by a majority of the members of our school. They use the methods of the old school, ignore the law of the similars, and when criticised for their measures of practice, declare that the critic is simply "raising the potency question."

This journal is constantly condemned as a high-potency journal, and the impression spread abroad that cures made with low potencies would not be admitted to its pages.

Of course, this is a stupid misapprehension, unworthy of any physician who has intellect enough to study symptomatic materia medica.

The plain intent of this journal, the motive for its foundation, was simply to teach the law of the similars, to demonstrate its success in practice, to inculcate its persevering application, to discourage the use of drugs in massive doses sufficient to produce ordinary gross physiological effects, and to encourage students in the acquirement of this knowledge that would enable them to become successful healers of the sick without the risks of injury and loss of patients common with all other methods of treatment.

The potency question cannot be settled, because there have not yet arisen sufficient data upon which to base a conclusion. It is folly to discuss it when experience with it varies so widely, resulting opinion so at variance, and, withal, there is so much that is unknown. It can only be approached in the calm, unbiassed spirit of investigation that seeks only the truth for the truth's own sake. This was the position held by the late Dr. Lippe during his long career, and which caused him to discourage all dispute concerning the potency which he regarded, in the present state of our knowledge, as superfluous and injurious, and as giving eclectics a convenient screen to shield themselves when their unhomœopathic methods of practice were attacked.

